# Ansamitocin P3-Loaded Gold-NanoCage Conjugated with Immune Checkpoint Inhibitor to Enhance Photo-Chemo-Thermal Maturation of Dendritic Cells for Hepatocellular Carcinoma

**DOI:** 10.3390/polym13162726

**Published:** 2021-08-15

**Authors:** Hung-Wei Cheng, Yu-Ling Ou, Chia-Chi Kuo, Hsin-Yi Tsao, Huai-En Lu

**Affiliations:** 1 Department of Materials Science and Engineering, National Yang Ming Chiao Tung University, Hsinchu 30010, Taiwan; mlb14756@gmail.com (H.-W.C.); annou831101@yahoo.com.tw (Y.-L.O.); d42172000@gmail.com (C.-C.K.); yvone880217@gmail.com (H.-Y.T.); 2 Bioresource Collection and Research Center, Food Industry Research and Development Institute, Hsinchu 300193, Taiwan

**Keywords:** gold nanocages, photothermal therapy, DCs maturation, immunotherapy, immune checkpoint inhibitor

## Abstract

Immunotherapy is a newly developed method for cancer treatment, but still generates limited response in partial patients for hepatocellular carcinoma (HCC) because the immunity cycle is limited by the tumor microenvironment (TME). Herein, we introduce multifunctional gold nanocages (AuNCs)-based nanocarriers with Ansamitocin P3 (AP3) loaded and anti-PDL1 binding (AP3-AuNCs-anti-PDL1) which can combine photothermal therapy, chemotherapeutic agent-triggered DCs maturation, and checkpoint immunotherapy in one platform. The AP3-AuNCs-anti-PDL1 using Avidin-biotin to bind anti-PDL1 on the surface of AP3-AuNCs showed specifically cellular targeting compared to AuNCs, which can increase the immune responses. The AP3-AuNCs+NIR-10 min exhibited the highly activated DCs maturation with two-fold higher than control+NIR, which can be attributed to the significant release of AP3. The results illustrated the synergistic effect of tumor-associated antigens (TAAs) and controlled AP3 release under near infrared (NIR) in triggering effective DCs maturation. Among them, AP3 release played the more important role than the TAAs under PTT in promoting T-cell activation. These results illustrate the promising potential of AuNCs-based nanocarriers combined with AP3 and the checkpoint inhibitors to strengthen the positive loop of immunity cycle.

## 1. Introduction

Immunotherapy has become a promising therapeutic strategy for treating cancer. Many immune treatments have now been developed to fight cancer via stimulating immune system to attack cancer cells, but currently a few trials are conducted for hepatocellular carcinoma (HCC) because of low patient response rate of immune checkpoint inhibitors (ICIs) which are associated with decreased tumor infiltration of CD8+ T cells and block dendritic cells (DCs) function in non-immunogenic tumor microenvironment [1,2,3,4,5,6,7,8]. To achieve effective immunotherapy, a series of functional stepwise pathways called the “cancer-immunity cycle” where the tumor-associated antigens (TAAs) presented on DCs surface has to be initiated [9]. There are many methods to induce the formation of TAAs by the immunogenic cell death (ICD), such as chemotherapy, radiation therapy, and photothermal therapy (PTT). Further, ICD can express the damage-associated molecular patterns (DAMPs) to stimulate DCs maturation and migration [10,11]. Among them, PTT was usually considered as the best candidate to selectively heat tumor region without damaging the normal cells compared to other treatments [12,13]. However, the maturation on the DCs through TAAs induced by PTT may be limited to achieving effective activation of T cells since the inhibition of tumor microenvironment (TME) and localized near infrared (NIR), which need to add inducer of DCs stimulation for converting immature DCs into mature DCs [14,15,16]. Therefore, it became an important issue to develop a nanocarrier with both chemotherapy and photothermal therapy contributing to mature DCs for increasing tumor infiltration of CD8+ T cells to raise the response of ICIs.

Several drugs or strategies have been adopted to mature DCs [17,18,19]. Recently, low-dose (noncytotoxic concentrations) chemotherapeutic agents as immune-adjuvant were reported to induce DCs maturation. Martin et al. further investigated the pharmacological effect of different class cytotoxic compounds, and found that microtubule-destabilizing compounds were able to induce DCs stimulation [20]. Among them, Ansamitocin P3 (AP3), a kind of microtubule-depolymerizing agent isolated from Nocardia, was identified as the most effective inducer of DCs maturation to induce a potent phenotypic and functional maturation for activating T cells at a lower dose. In addition, AP3 can enhance T-cell stimulatory capacity through up-regulation of maturation markers on the DCs and production of pro-inflammatory cytokines [20]. However, AP3 is hardly soluble in water because its solubility in pure water is only 20 mg/L under normal temperature and pressure. Therefore, it becomes an important issue to develop a nanostructure as a platform to integrate the AP3 and the PTT with ICI addition for improving the immune response.

**Scheme 1:**
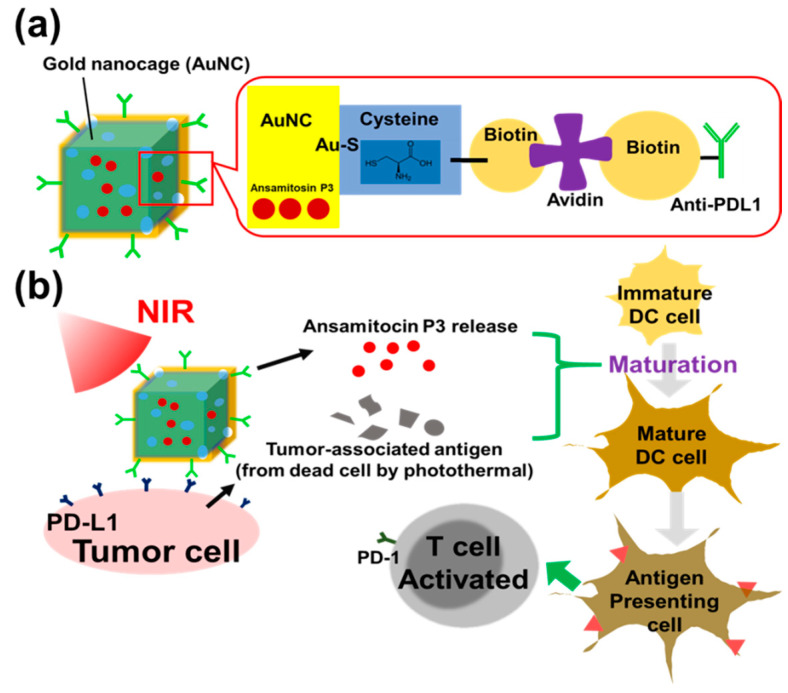
Schematic illustration of (**a**) component of multifunctional AuNCs-based immunanoparticle and (**b**) the synergistic mechanisms of both photo-chemotherapy and photo-thermal therapy to mature DCs under NIR irradiation.

Gold (Au) nanostructures have received wide attention because of their excellent optical properties, bio-inertness, and low cytotoxicity among all kinds of biocompatible nanoparticles [21,22,23]. In the study, to achieve both chemotherapy and PTT with enhanced permeability to spread and to eradicate wide-ranged malignant cells, a multifunctional AP3 loaded-AuNCs with anti-PDL1 targeting system (AP3-AuNCs-anti-PDL1) was designed to combine PTT, chemotherapeutic agent, and checkpoint immune therapy in one platform as shown in Scheme 1a. Under NIR irradiation at 808 nm, the AP3 loaded-AuNCs are used as photosensitizer to produce PTT for generating tumor-associated antigen and simultaneously release AP3 to stimulate maturation of DCs to boost the immune response. In addition, anti-PDL1 checkpoint inhibitor can be used to selectively target the tumor cell which shows high expression of PDL1 on the surface to enhance the accuracy of PTT. The mechanisms and the advantages of the AP3-AuNCs-anti-PDL1 are also illustrated in Scheme 1b. First, anti-PDL1 is capable of blocking PDL1 on the surface of liver cancer cells (Hep55.1c) to increase the accuracy of PTT and enhance immune response. Second, PTT produced by AuNCs under the NIR exposure can not only induce apoptosis but also release AP3 at the same time to promote DCs maturation. Overall, this AuNCs-based nanocarrier with the anti-PDL1 as a platform to produce the TAAs and control the AP3 release for stimulating DCs maturation, lead to the activation of T cell and specific targeting of the tumor cells to create an immunogenic TME. The AP3-AuNCs-anti-PDL1 with targeting and PTT can achieve multiple stimuli to maturate the DCs, and thus the response rate of immune checkpoint inhibitors can be much enhanced. This strategy can provide more effective DCs maturation to increase the immuno-therapeutic efficacy and is worth further investigation to explore the actual in vivo effect in different tumor models.

## 2. Materials and Methods

### 2.1. Materials

Ethylene glycol, silver nitrate (AgNO_3_), PVP (powder, average Mw~ 29,000), Na_2_S 9H_2_O, HAuCl_4_·3H_2_O, sodium chloride, N-hydroxysuccinimide (NHS, 98%), N-(3-dimethylaminopropyl)-N’-ethylcarbodiimide hydrochloride (EDC, 98%), L-cysteiene (CYS), ansamitocin P3 were purchased from Sigma Aldrich Chemical Co (Burlington, NJ, USA). Rhodamine B used to label the nanocarriers for visualization under a fluorescence microscope was purchased from Ocean NanoTech (SAN DIEGO, CA, USA). Anti-PDL1 antibody, 4′,6-diamidino-2-phenylindole (DAPI), phalloidin, Rat IgG antibody (FITC) were purchased from Thermo Fisher Scientific (Waltham, MA, USA). Interleukin 4 (IL-4) was purchased from Sino (Wayne, MI, USA). Alexa Fluor^®^ 488 Goat Anti-Mouse IgG, F(ab’)2 fragment specific, was purchased from Jackson ImmunoResearch (West Grove, PA, USA).

### 2.2. Methods

#### 2.2.1. Preparation of Gold Nanocages (Auncs)

The procedure of synthesizing AuNCs followed Xia’s group publication reported in 2007 [24]. The entire process was divided into two parts including synthesis of sliver nanocubes (AgNCs) and AuNCs. For AgNCs formation, briefly, the oil bath was pre-heated to 150 °C and then four clean reaction vials containing ethylene glycol (EG, 6 mL) were put into the oil bath for 1 h. After that, two Na_2_S EG solutions (3 mM, 70 μL, and 100 μL) were pipetted into the vials, respectively, followed by waiting for 8 min. Then PVP EG solution (0.18 M, 1.5 mL) and AgNO_3_ EG solution (0.28 M, 0.5 mL) were pipetted into the four vials, followed by waiting for 15 min. After the reaction vials cooled down to room temperature, the products were washed using acetone and spun down at 2000 g for 30 min. Then, the products were again washed with deionized water for three times and centrifuged at 9000× *g* for 10 min. AgNCs were dispersed in deionized (DI) water (4 mL) and stored at 4 °C until use. For synthesis of AuNCs, AgNCs solution and PVP solution (9 mM, 10 mL) were pipetted into a round-bottom flask and then titrated HAuCl_4_ solution (0.2 mM) was added to the mixture at a rate of 10–15 mL/h. For a desirable LSPR peak of AuNCs, color changes and UV-visible spectrum (Evolution 300, Thermo Fisher Scientific, Waltham, MA, USA) were both observed. After the reaction was stopped, excessive sodium chloride (NaCl) was added to the mixture, and then the supernatant was collected and washed three times at 11,000 rpm, 10 min. AuNCs were collected in DI water at 4 °C.

#### 2.2.2. Synthesis of Cys-Biotin-Avidin-Biotin-Anti-PDL1

The synthesis process of L-cysteine (Cys)-Biotin-Avidin-Biotin-anti-PDL1 can be divided into two pathways. First, Cys-Biotin and Biotin-anti-PDL1 were synthesized. The Sulfo-NHS-Biotin (10 mM, 25 μL) was added to Cys (1 mg/mL) and anti-PDL1 (1 mg/mL) in the ice for 2 h in the dark, respectively. To purify the product, Cys-Biotin and anti-PDL1-Biotin were centrifuged at 1000 *rcf.* for 2 min and repeated three times. The obtained Cys-Biotin-Avidin-Biotin-anti-PDL1 was suspended in deionized water at 4 °C.

#### 2.2.3. Synthesis of AP3-Auncs-Cys-Biotin-Avidin-Biotin-Anti-PDL1 (AP3-AuNCs-anti-PDL1)

Both the Ansamitocin P3 (AP3) ethanol (EtOH) solution (50 ppm, 120 μL) and the Cys-Biotin-Avidin-Biotin-anti-PDL1 (1000 ppm, 9 μL) were added to 3 mL AuNCs (50 μg/mL). The mixed solution was reacted for 12 h at 4 °C. To remove the unloaded AP3 and Cys-Biotin-Avidin-Biotin-anti-PDL1, the mixed solution was added to a dialysis tubing cellulose membrane with cut off molecule weight of 14,000. The dialysis bags were dialyzed in 6 mL DI water at room temperature for 30 min. Then the DI water was changed and dialyzing was repeated four times. The obtained AP3-AuNCs-anti-PDL1 was suspended in DI water at 4 °C.

#### 2.2.4. Characterization of AP3-Auncs-Anti-PDL1

The morphology of AgNCs and AuNCs were characterized using scanning electron microscopy (SEM; JSM-6700-F, JEOL, Tokyo, Japan) and transmission electron microscopy (TEM; JSM-2100, JEOL, Tokyo, Japan). For SEM observation, the sample solution was dropped on silicon wafers, and dried in a vacuum desiccator at room temperature. For TEM observation, the sample solution was dropped on a carbon-coated copper grid. Residues of the droplet were removed and then the copper grid was dried in a vacuum desiccator at room temperature. The zeta potential and particle size of nanocages in DI water, 80% EtOH, and 99% EtOH solvent were measured by electrophoretic light scattering and dynamic light scattering (ELS and DLS; Delsa Nano C particle analyzer, Beckman Coulter, Brea, CA, USA). In order to prove the AP3 were successfully loaded in the AuNCs at different solvents, the chemical state was analyzed by using Fourier transform infrared spectroscopy (FTIR, spectrum 100, PerkinElmer). To confirm the conjugation of anti-PDL1, Alexa Fluor^®^ 488 Goat Anti-Mouse IgG, F(ab’)2 fragment specific was added to the AP3-AuNCs-anti-PDL1 for 1 h and washed three times. The results were observed using fluorescence microscopy (D-eclipse C1; Eclipse, TE2000-U, Nikon, Tokyo, Japan).

#### 2.2.5. Photothermic Effect of Aqueous AP3-Auncs

The temperature of AP3-AuNCs solution (AuNCs, 50 μg/mL) under 808 nm laser exposures (1 W/cm^2^) and the temperature elevation plot was recorded by a thermal couple which operated at 30 s interval for a 15-min period. The photothermal conversion efficiency (PTCE) of the AuNCs and AP3-AuNCs was determined according to the following formula (Equation (1)) [25,26]:

(1) $$\eta =\frac{hs\left(T_{Max}-T_{Surr}\right)-Q_{Dis}}{I\left(1-10^{-A808}\right)}$$

where *h* is the heat transfer coefficient, *s* is the surface area of the container. *T_Max_* − *T_Surr_* is the temperature change of the AP3-AuNCs solution at the maximum steady environmental temperature, *I* is the power of the laser, A808 is the absorbance of AP3-AuNCs at 808 nm, and *Q_Dis_* expresses heat dissipated from light absorbed by the solvent and the container.

#### 2.2.6. Drug-Loading Ability and in Vitro Drug Release

Loading capacity and encapsulation efficiency (EE) of AP3-AuNCs were determined using liquid chromatography-mass spectrometry (LC-MS) [27]. In brief, after dialysis of the AP3-AuNCs, the DI water out of the dialysis bags was collected and the AP3 of the DI water was quantified using LC-MS. The loading capacity and EE of the Ansamitocin P3 in the AP3-AuNCs were calculated as follows (Equations (2) and (3)):

(2) $$Loading\;capacity\left(\frac{w}{w}\right)=\frac{A}{B}=\frac{D-C}{B}$$

where *A* is the mass of the carried drug, *B* is the mass of the entire AP3-AuNCs, *C* is the mass of the drug not carried by AP3-AuNCs and quantified by LC-MS, *D* is the total mass of the added drug, and

(3) $$EE\;\%=\frac{E}{F}\times 100\;\%=\frac{F-G}{F}\times 100\;\%$$

where *E* is the amount of carried drug, *F* is the total amount of drug in feeding formula, and *G* is the amount of drug which stayed in solvent out of the dialysis bags and quantified by LC-MS.

For the drug release test, AP3-AuNCs (50 μg/mL, 0.5 mL) was added to dialysis tubing cellulose membrane with a cut off molecular weight of 14,000. The dialysis bags were dialyzed in DI water (2 mL) under 808 nm laser exposure (1 W/cm^2^) for 10 min and the incubation medium were collected at predetermined time points. The amounts of released drug were quantified using LC-MS method. All experiments were performed in triplicate.

#### 2.2.7. Cell Culture

Hep55.1c (RRID: CVCL_5766) mouse liver cancer cell line was donated by China Medical University, Taiwan. Hep55.1c was maintained in DMEM medium supplemented with 10% fetal bovine serum (FBS) and 1% penicillin-streptomycin. Bone marrow cells which were extracted from thigh bones of 6–8 weeks C57BL/6 WT mice were induced to differentiate to DCs in 5 to 6 days culture in RPMI 1640 medium supplemented with 10% FBS, 1% sodium pyruvate, 1% nonessential amino acids, 1% penicillin/streptomycin, GM-CSF (20 ng/mL), and IL-4 (10 ng/mL). The cells were cultured in complete medium at 37 °C in a humidified atmosphere with 5% carbon dioxide in air.

#### 2.2.8. Determination of Targeting Efficiency of AP3-Auncs-Anti-PDL1

In order to observe the target behavior between AP3-AuNCs-anti-PDL1 and Hep55.1c cells, fluorescent staining technique was used to demonstrate AuNCs targeting. The fixed Hep55.1c cells’ F-actin structure and nuclei were stained by green fluorescent phalloidin and blue fluorescent 4′,6-diamidino-2-phenylindole (DAPI), respectively. Rhodamine B (RhoB) was conjugated with AuNCs that made nanocarriers highly visible under a fluorescence microscopy. A total amount of 3 × 10^5^ Hep55.1c cells were seeded and grew on glass coverslips in the 6-well plates for 24 h. The cells were then incubated with RhoB-labeled AP3-AuNCs or AP3-AuNCs-anti-PDL1 for different periods of time. Afterward, Hep55.1c cells were washed with PBS three times and fixed for 30 min with 3.7% formaldehyde. Cellular permeability was performed with 0.25% Triton X-100 for 15 min. Finally, after cell staining with F-actin and DAPI for 1 h and 30 min, respectively, the cells were mounted on a clean glass slide by mounting medium and observed under fluorescence or confocal microscopy (D-eclipse C1; Eclipse, TE2000-U, Nikon, Tokyo, Japan).

#### 2.2.9. Cytotoxicity Assay of Hep55.1c Treated with Photothermal Therapy

A total of 1 × 10^5^ Hep55.1c cells were cultured in a 96-well plate for 24 h prior to the cytotoxic test. AP3-AuNCs (50 μg/mL) were added to the well and incubated for 24 h. After that, the Hep55.1c cells were treated with 808 nm diode laser (1 W/cm^2^; 2, 5, 10, 15 min) or nothing as control. After the treatment, the cells were maintained for further 1 day. Then, the MTS reagent (10%) containing medium was added to each well for 2 h and incubation, and the optical absorbance was measured at 490 nm using an ELISA reader (DV990BV4, GDV, Milan, Italy). On the other hand, to evaluate the AP3-AuNCs cell cytotoxicity, after 1 × 10^5^ Hep55.1c cells were cultured in a 96 well plate for 24 h, the cells were incubated with FBS-free medium containing various concentrations of AuNCs (0, 1, 10, 20, 40, 80, 100 μg/mL) for 24 h. Then, the MTS reagent (10%) containing medium was added to each well and incubated for 2 h and the optical absorbance was measured at 490 nm using an ELISA reader (DV990BV4, GDV, Milan, Italy).

#### 2.2.10. Analysis of Mature Dcs Surface-Marker Expression

The method refers to the protocol paper of Alka et al. [28]. After 6 days of incubation, non-adherent primary bone marrow dendritic cells (BMDC) were collected by gently pipetting them with culture medium. For in vitro DCs stimulation experiments, we cocultured AP3, AuNCs, and AP3-AuNCs (AuNCs = 50 μg/mL, AP3 = 1 μg/mL) with 2 × 10^5^ bone-derived DCs from C57BL/6 mice for 20 h. For transwell (Corning Costar^®^, New York, NY, USA) experiments, residues of Hep55.1c cells after photothermic treatment with AP3-AuNCs were also added to the 10^6^ DCs culture for 20 h. The DCs stained with anti-CD11c-FITC (RUO), anti-CD80-PE (16-10A1), and anti-CD86-PE (GL1) were assessed by flow cytometry (Novocyte^®^, ACEA biosciences, San Diego, CA, USA).

## 3. Result and Discussion

### 3.1. Synthesis of Silver Nanocubes (Agncs) and Gold Nanocages (Auncs)

AuNCs were prepared according to a robust protocol through two steps [24]: sulfide-mediated silver nucleation and the subsequent reconstruction by the galvanic replacement reaction as the following equation (Equation (4)).

3 Ag_(s)_ + AuCl^4−^_(aq)_ → 3 Ag^+^_(aq)_+ Au_(s)_ +4 Cl^−^_(aq)_ (4)

**Figure 1:**
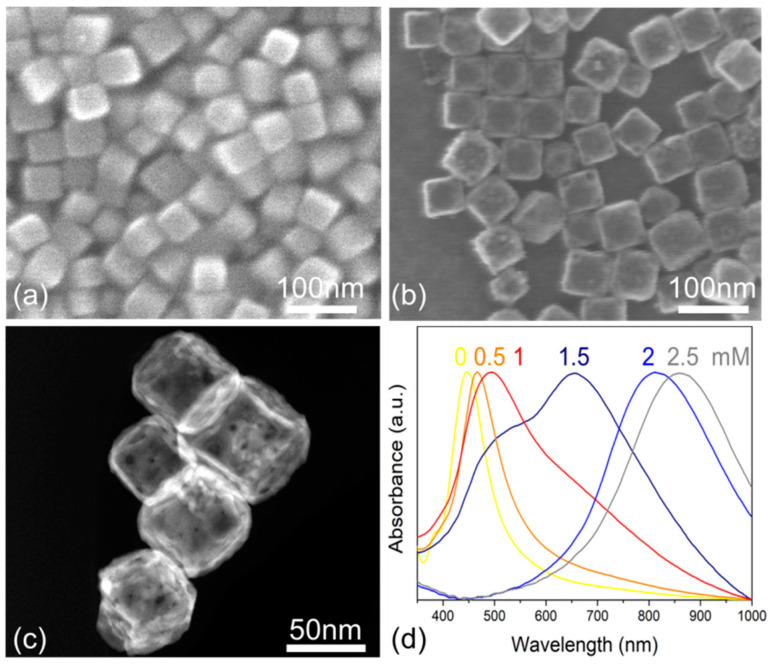
Images and optical properties of AgNCs and AuNCs. (**a**) SEM image of AgNCs, which showed approximately 50 nm in edge length. (**b**) SEM image and (**c**) TEM image of AuNCs showing a hollow and porous structure. (**d**) UV-visible-NIR absorbance spectra of AgNCs and AuNCs by adding different volumes of HAuCl_4_ solution (0, 0.5, 1, 1.5, 2, 2.5 mM).

The size of the obtained AgNCs was observed to be around 50 nm in edge length measured using SEM in Figure 1a. Subsequently, AuNCs were formed in a galvanic replacement reaction and the original square shape was retained as shown in Figure 1b. A high-magnification STEM image displayed a hollow structre with porous walls in Figure 1c. During the galvanic replacement reaction, with increasing the volume of HAuCl_4_ solution, the absorbance peak alternated from 440 nm to the near infrared (NIR) range as shown in Figure 1d, which can be evidenced and monitored through serial color changes using UV-visible spectroscopy (Figure S1).

### 3.2. Characterization of Ansamitocin P3-Loaded Gold Nanocages (AP3-Auncs)

A photo-sensitive AP3-AuNCs was enshrouded with L-cysteine (CYS) through gold-sulfur interaction to conjugate the biotin to bind the anti-PDL1. The solubility of AP3 is quite low (water solubility = 20 μg/mL) in aqueous medium but ethanol is a better solvent for dissolving AP3 (1 mg/mL). Therefore, the AP3-AuNCs synthesized in distilled deionized water (ddw), 80% ethanol (EtOH) solution, and 99% EtOH were investigated [29,30].

**Figure 2:**
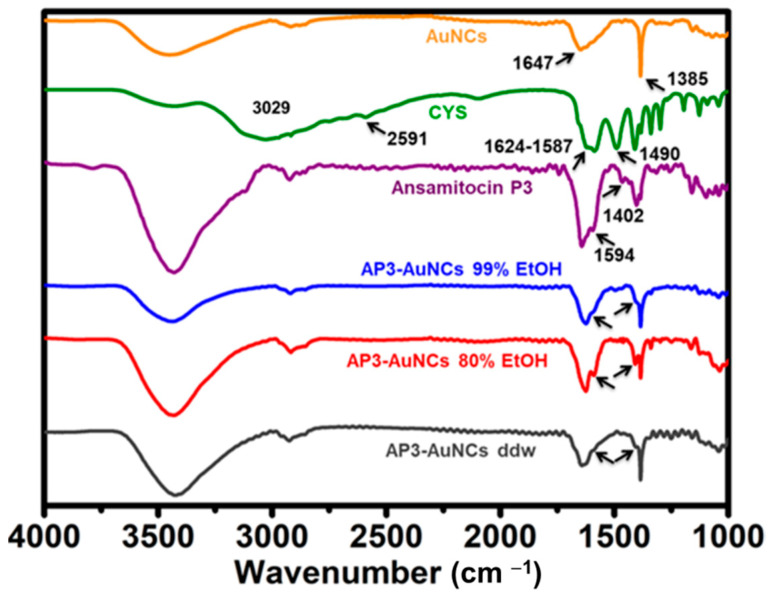
Fourier transform infrared spectra (FTIR) of AuNCs, CYS, AP3, AP3-AuNCs in ddw, 80% EtOH and 99% EtOH.

For verifying the Ansamitocin P3 loading, Fourier transform infrared spectra (FTIR) were used to verify the components of AP3-AuNCs conducted in the three solvents as shown in Figure 2. The AuNCs showed the major characteristic peak at 1647 cm^−1^ (C=O vibration) and 1385 cm^−1^ (C-N vibration) because of the existence of the PVP on the surface of AuNCs with its skeletal formula shown in Figure S2a. The spectrum of L-cysteine (CYS) presented a broadband at 3029 cm^−1^ (NH_3_^+^ vibration), a peak at 2591 cm^−1^ (S-H vibration), two peaks overlapped in 1624-1587 cm^−1^ (C=O vibration and NH_3_^+^ vibration), and a peak at 1490 cm^−1^ (NH_3_^+^ bending) [31]. However, the spectra of the AP3-AuNCs did not present the major peaks of CYS corresponding to NH3^+^ vibration, S-H vibration, and NH3^+^ bending possibly because of the gold-thiol interaction with AuNCs. The major peaks of AP3 indexed around 1594 cm^−1^ and 1402 cm^−1^ in Figure 2 appeared in all the samples. However, AP3-AuNCs synthesized in 80% EtOH exhibited the stronger AP3 peaks. In addition, the AP3-AuNCs in 80% EtOH displayed the stable morphology with a particle size distribution of approximately 120 nm and weak negative zeta potential of −9.46 mV (Table S1). Therefore, AP3-AuNCs conducted in 80% EtOH solution was selected for further investigation.

**Figure 3:**
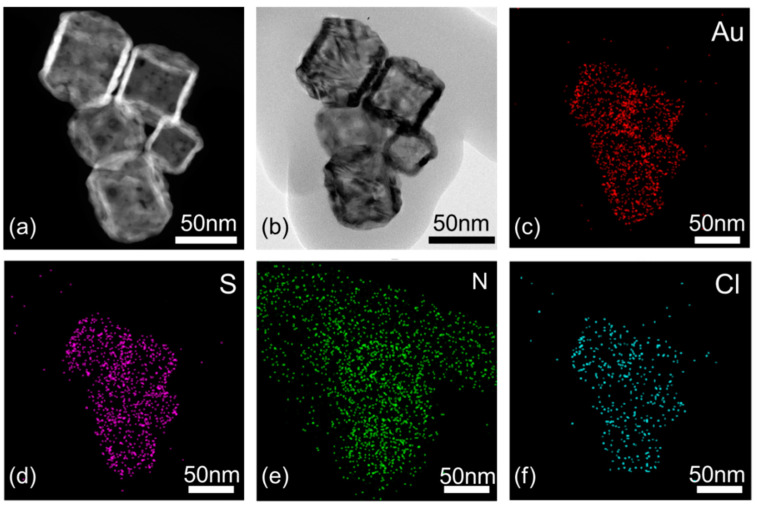
(**a**) STEM image, (**b**) TEM image and (**c**–**f**) elemental mapping of Au, S, N, and Cl AP3-AuNCs.

To confirm the morphology of AP3-AuNCs, TEM with elemental mapping of AP3-AuNCs using EDS was shown in Figure 3. The AuNCs were completely covered by the CYS to reduce AP3 leakage in Figure 3a. Further, the AuNCs mapping (Figure 3b) showed a clear hollow and square structure. The elemental mapping of sulfur (S) and nitrogen (N) overlapped the area of Au in Figure 3c–e was attributed to the AuNCs with the CYS coated. Moreover, the chlorine (Cl) in Figure 3f indicated the AP3 drug distributed in the inner layer of AuNCs. From the results, it can be confirmed that the AP3 was successfully loaded in the AuNCs and the AuNCs was covered by the CYS to reduce the leakage of AP3. The encapsulation efficiency (EE) and loading capacity (µg AP3/µg AP3-AuNCs) of AP3 were measured around 49% and 2.04%, respectively.

### 3.3. Heating Efficiency and AP3 Release of AP3-Auncs

**Figure 4:**
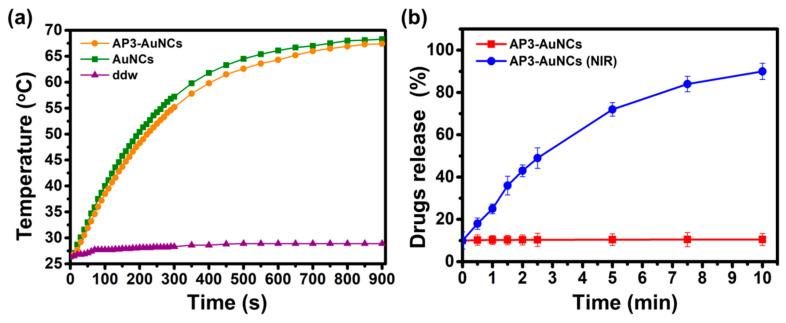
(**a**) Profile of temperature increase for 50 µg/mL AuNCs and AP3-AuNCs upon 808 nm laser irradiation at a power density of 1 W/cm^2^. (**b**) Cumulative AP3 release from AP3-AuNCs with NIR irradiation time.

As a photothermal treatment was applied to induce death of heat-sensitive cancer cells, AP3 was simultaneously released outside the tumor cell to activate the immature DCs in the tumor microenvironment. To evaluate the photothermal effect of AP3-AuNCs, the temperature increase of AP3-AuNCs with irradiation time under 808 nm laser irradiation at a power density of 1 W/cm^2^ was monitored. As shown in Figure 4a, AuNCs and AP3-AuNCs displayed significant temperature increase to 43 °C in 150 s and 180 s, respectively, indicating that the heating efficiency of both AuNCs and AP3-AuNCs was barely influenced by CYS coating and AP3 loading. In contrast, only a slight temperature increase from 25 °C to 29 °C was observed from the control of ddw. The photothermal stability of AP3-AuNCs and AuNCs was further investigated. As shown in Figure S3, the morphology of both nanoparticles still presented a cubic shape after the samples were repeated three times with a total irradiation time of 45 min. In addition, the PTCE of AuNCs and AP3-AuNCs were calculated about 65.19% and 58.54%, respectively. The results showed that the PTCE of both AuNCs and AP3-AuNCs was higher than gold nanorods (GNRs) and organic nanoparticle under the 808 nm-laser. This illustrated that AP3-AuNCs can facilitate the photothermal ablation of cancer cells [32,33]. Further, AP3 drug release from AP3-AuNCs can be further triggered and promoted by using 808 nm laser irradiation at 1 W/cm^2^. The results indicated that AP3-AuNCs presented a sustained AP3 release with time and reached up 90% in 10 min in Figure 4b. In contrast, the AP3 release was negligible without NIR triggering in control group, showing the AP3 remaining in AuNCs at room temperature.

### 3.4. Characteristic of Anti-PDL1-Targeted AP3-Auncs

**Figure 5:**
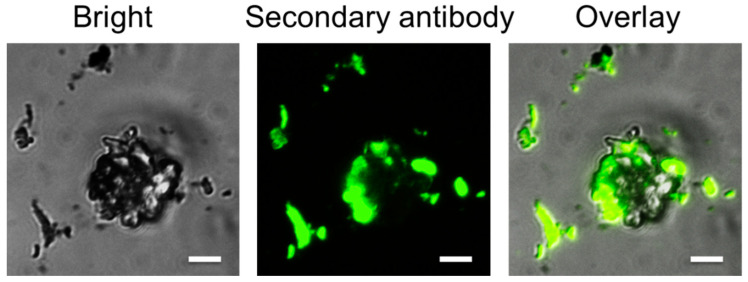
Immunofluorescence of AP3-AuNCs-anti-PDL1. (Scale bar = 1 μm).

One of the important concerns of cancer therapy is to selectively deliver therapeutic agents to the targeted cells. Programmed death-ligand 1 (PDL1) has been exclusively overexpressed on cell surface of numerous metastatic cancer cells so it can also be considered as a reliable surface-binding site for targeted therapy to increase the activities of immune responses [34,35]. As a result, the surface of AuNCs was grafted with CYS-biotin to conjugate the anti-PDL1. To ensure the conjugation and direction of anti-PDL1 to present its targeting function, the immunofluorescence for targeting the fragment of F(ab’)2 was performed in Figure 5 which confirmed that the anti-PDL1 has been successfully bonded on the AuNCs surface via Biotin. In addition, the direction of the anti-PDL1 Fab is outward to prove its specific function to tumor cells.

### 3.5. Cellular Targeting Efficiency of AP3-Auncs-Anti-PDL1

**Figure 6:**
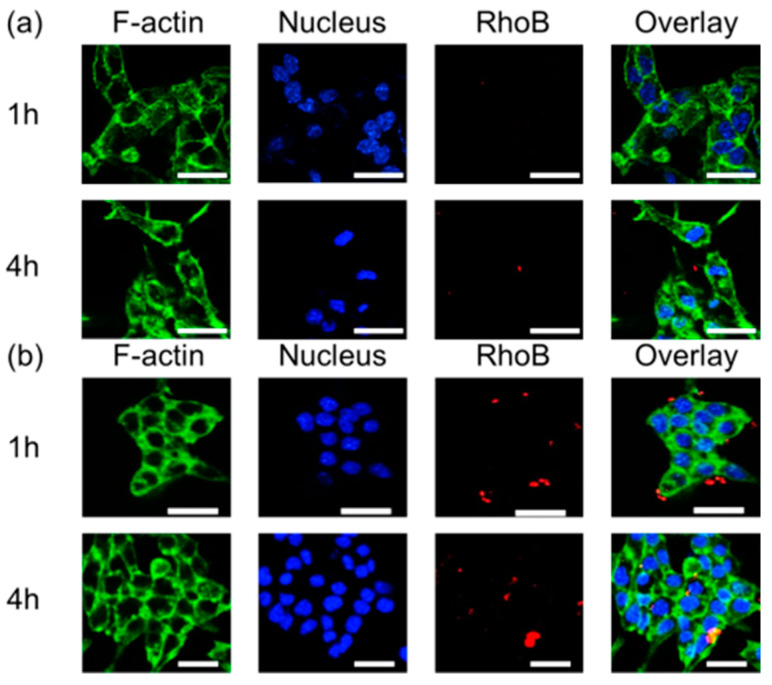
The cell association evaluation of (**a**) non-targeted RhoB-labeled AP3-AuNCs and (**b**) RhoB-labeled AP3-AuNCs-anti-PDL1 with 1 h and 4 h incubation using fluorescent staining. The f-actin, the nucleus of cells, and the RhoB-labeled AP3-AuNCs/RhoB-labeled AP3-AuNCs -anti-PDL1 were labeled as green, blue, and red colors, respectively (Scale bar = 10 μm).

Hepatocellular carcinoma (HCC), also known as liver cancer, exhibited high postoperative recurrence and resistance to chemotherapeutic drugs, a novel and effective therapy for HCC is thus urgently needed. The highly immunosuppressive microenvironment of HCC tumors suggested that HCC may be suitable for immunotherapy, and anti-PDL1 immune checkpoint inhibitors showed to be promising for the treatment of HCC [36,37,38]. To evaluate internalization of nanocages, rhodamine B (RhoB) was labeled to the AP3-AuNCs for fluorescent detection. The negligible red fluorescence was observed for the cells treated with RhoB-labeled AP3-AuNCs for 1 h and 4 h in Figure 6a. To enhance AP3-AuNCs targeting, RhoB-labeled AP3-AuNCs were conjugated with anti-PDL1 antibody. The results in Figure 6b showed that the targeting efficiency of RhoB-labeled AP3-AuNCs-anti-PDL1 to HCC was significantly higher as compared to that of AP3-AuNCs without anti-PDL1 within 1-h incubation period and more obvious after 4-h incubation in Figure 6b. The results demonstrated that RhoB-labeled AP3-AuNCs-anti-PDL1 could rapidly and effectively target to Hep55.1c cell at a very short time through antibody–antigen specific interaction.

### 3.6. Cytotoxicity Assay and Photothermal Therapy of AP3-Auncs

**Figure 7:**
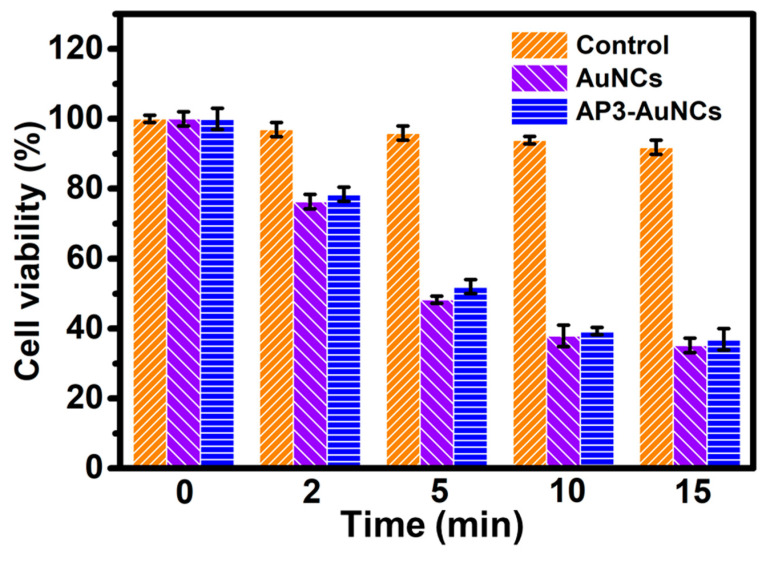
In vitro cell viability for AP3-AuNCs and AuNCs post 24 h incubation induced by PTT. For PTT effect, the cells were irradiated under 808 nm diode laser (1 W/cm^2^) for 2, 5, 10, and 15 min. Data are expressed as mean ± SD, *n* = 3.

To investigate the cytotoxicity of AP3-AuNCs and AuNCs, Hep55.1c cells were incubated with different concentrations of AP3-AuNCs and AuNCs for 24 h in dark. Cell viability test in Figure S4 showed that the AuNCs and AP3-AuNCs displayed negligible cytotoxicity with the incubation of 100 μg/mL AuNCs, indicating that both materials displayed good biocompatibility under proper dose utilization. When the heating temperature was controlled at 41–45 °C, nanoparticle-induced hyperthermia can lead to tumor cell death without causing serious injury to adjoining normal tissues. The phototherapeutic capabilities of AuNCs and AP3-AuNCs to Hep55.1c cells were further studied in dark or under 808 nm laser irradiation (1 W/cm^2^). The cell viabilities of Hep55.1c cells were not significantly affected by AuNCs and AP3-AuNCs (concentration = 50 μg/mL) under dark condition in Figure 7. When the Hep55.1c cells were treated with NIR for 2 min, the cell viabilities were between 75 and 80% because of poor thermal efficiency. However, as the Hep55.1c cells were treated for 5, 10 and 15 min, the cell viabilities were reduced to below 50%. In contrast, minimal effect was observed without AP3-AuNCs even though the cells were irradiated under 808 nm for 10 min. These results demonstrated that AuNCs displayed excellent photothermic effect under a low-power density of 1 W/cm^2^ at a low-concentration of AuNCs compared to Au-nanostructured nanoparticles [39,40,41]. More importantly, both AP3-AuNCs and AuNCs displayed similar cell viabilities, proving that AuNCs with AP3 loaded showed no interference for NIR photothermic effect.

### 3.7. In Vitro Dcs Maturation Efficiency of AP3-Auncs

**Figure 8:**
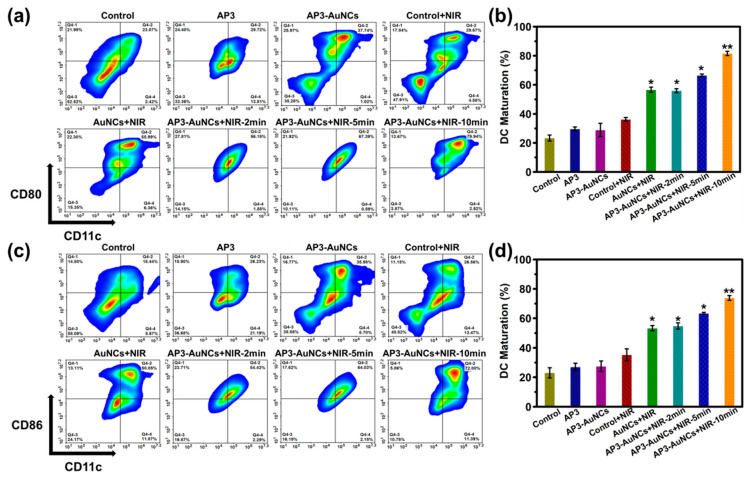
Flow cytometry expression of (**a**–**b**) CD11c CD80 (**c**–**d**) CD11c CD86 on mature DCs surface after feeding AP3, AuNCs and AP3-AuNCs. Two-tail Student’s test was used. * *p* < 0.05, ** *p* < 0.01.

For efficient T-cell stimulation, mature DCs should express or upregulate T-cell-costimulatory molecules. CD11c^+^ is transmembrane protein that is expressed on the immature DCs and mature DCs. Mature DCs could express up-regulation of co-stimulatory molecules (CD80 and CD86) on the surface. As shown in Figure 8, DCs maturation induced by AP3 and PTT was further assessed for the untreated Hep55.1c cells added with AuNCs, AP3 and AP3-AuNCs in the transwell systems by flow cytometry. The DCs maturation is limited for AP3 group only, but an obvious increase in DCs maturation was observed in AP3-AuNCs because a higher dosage of AP3 could be delivered to the DCs using AuNCs. On the other hand, as the Hep55.1c were treated by PTT using AuNCs or AuNCs-AP3, the AP3-AuNCs+NIR-10 min exhibited the highly activated DC maturation with 2–2.5 fold higher than AP3-AuNCs or control+NIR. Further, to evaluate the combination of AP3 dose and TAAs, NIR treatment with various time was applied. Figure 8b showed that CD80 was measured as 55.98 ± 1.38, 66.54 ± 0.9, and 81.58 ± 1.58% under PTT treatment for 2, 5, and 10 min of PTT, respectively. In addition, CD86 was also measured as 54.81 ± 2.12, 63.38 ± 0.7, and 73.85 ± 1.61% in 2, 5, and 10 min of PTT in Figure 8d, respectively. As compared to DCs maturation in Figure 8 with the temperature profile of AP3-AuNCs and cumulative AP3 release upon 808 nm laser irradiation at a power density of 1 W/cm^2^ in Figure 4, it was noted that the AP3-AuNCs was irradiated with NIR for 2 min, the temperature up to 43–45 °C is enough for killing the tumor cells (20% in Figure 7) to produce TAAs but AP3 release is about 40–45%. With increasing exposure time to 10 min at PTT, AP3-AuNCs+NIR-10 min exhibited the activated DC maturation with a ~30% increase compared to AP3-AuNCs+NIR-2min, which can be attributed to the significant release of AP3 to 90%. The results illustrated the importance of significant AP3 release in triggering the effective DCs maturation and then T-cell activation under the combination treatment of post-PTT and AP3. Compared with photothermal or photodynamic nanoparticle carrying immune-adjuvant agent [18,42], AP3-AuNCs displayed high DCs maturation in vitro, which can be attributed to the synergistic effect of generating tumor-associated antigens and simultaneously releasing AP3 to stimulate DCs.

## 4. Conclusions

In this study, a multifunctional gold nanocages (AuNCs)-based nanocarrier was developed for hepatocellular carcinoma (HCC) by nanoparticle-assisted photothermal therapy (PTT), chemotherapeutic agent-triggered dendritic cells (DCs) activation, and checkpoint immune therapy. By using this gold nanocages (AuNCs)-based nanocarrier, the hydrophobic AP3 can be closely encapsulated to promote the release as a potent inducer of phenotypic and functional maturation of DCs under the NIR exposure to significantly trigger the maturation of DCs and immunity promotion. This AuNCs-based nanocarrier with the anti-PDL1 as a platform to produce the TAAs and control the AP3 release for stimulating DCs maturation, leading to the activation of T cell and specific targeting of the tumor cells to create an immunogenic tumor microenvironment. Moreover, the achievement illustrates the promising in HCC cancer therapy using AP3-AuNCs-anti-PDL1 in vivo and the clinical applications.

## Data Availability

The data presented in this study are available on request from the corresponding author.

## Supplementary Materials

The following are available online at https://www.mdpi.com/article/10.3390/polym13162726/s1, Figure S1: HAuCl 4 solution (0, 0.5, 1, 1.5, 2, 2.5 mM) were used to titrate Ag nanocubes suspension and brilliant color was changed during Ag Aualloy formation; Figure S2: The structure of PVP and Anasmitocin P3; Figure S3: The morphology of AuNCs and AP3 AuNCs under 808 nm irradiation; Figure S4: Evaluation of cell viability of Hep55.1c cells after incubation with various concentrations of AuNCs and AP3-AuNCs in dark for 24 h, respectively. Error bars are expressed standard error with *n* = 3; Table S1: Characterization of the AP3-AuNC constructed in different solvents.
