# Supplementary material for: Ansamitocin P3-Loaded Gold-NanoCage Conjugated with Immune Checkpoint Inhibitor to Enhance Photo-Chemo-Thermal Maturation of Dendritic Cells for Hepatocellular Carcinoma
Source: Polymers (Basel). 2021 Aug 15;13(16):2726. doi: 10.3390/polym13162726 (PMC8398096; doi:10.3390/polym13162726)
Supplement: Supplementary file 1 [file polymers-13-02726-s001.zip › polymers-1309683-supplementary.pdf]

Supporting Information:

Ansamitocin P3-loaded Gold-NanoCage Conjugated with Immune Checkpoint Inhibitor to Enhance Photo-Chemo-Thermal Maturation of Dendritic Cells for Hepatocellular Carcinoma

Hung-Wei Cheng, Yu-Ling Ou, Chia-Chi Kuo, Hsin-Yi Tsao, and Huai-En Lu

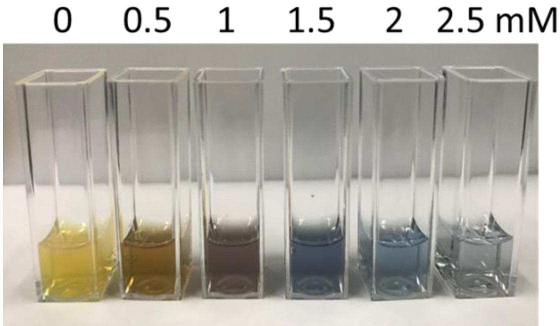

Figure S1. $HAuCl_4$ solution (0, 0.5, 1, 1.5, 2, 2.5 mM) were used to titrate Ag nanocubes suspension and brilliant color was changed during Ag-Aualloy formation.

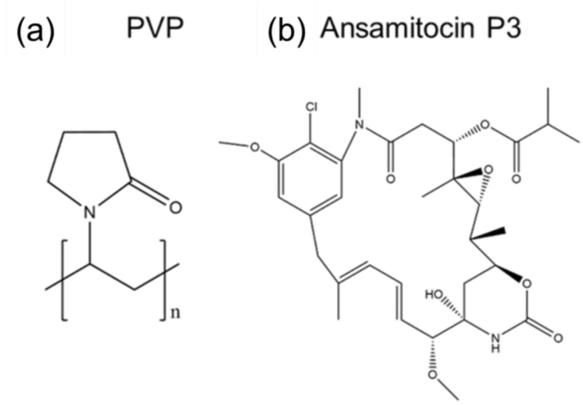

Figure S2. The structure of PVP and Anasmitocin P3

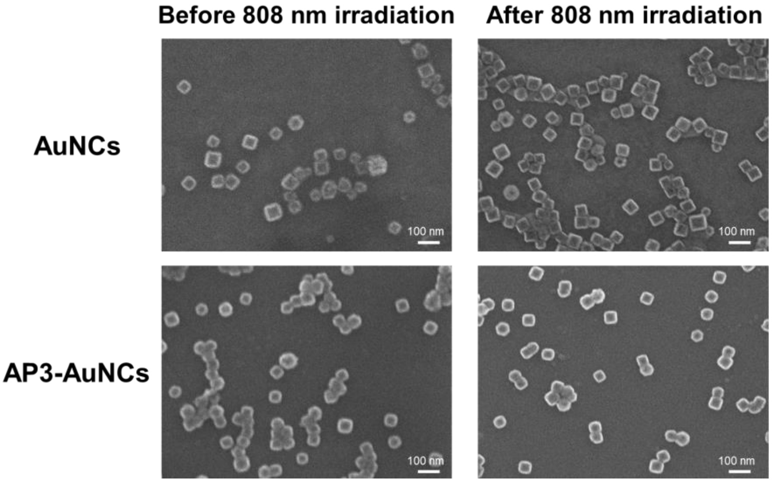

Figure S3. The morphology of AuNCs and AP3-AuNCs under 808 nm irradiation.

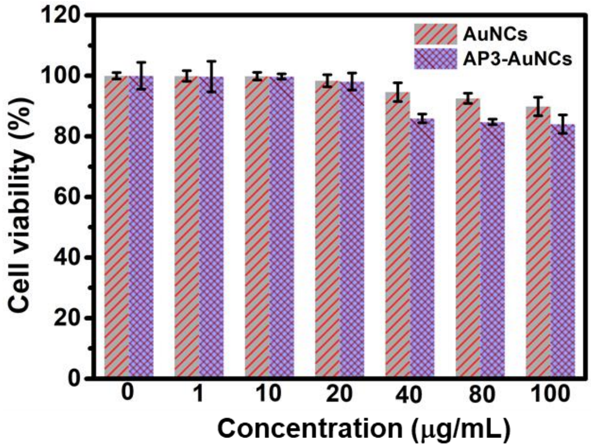

Figure S4. Evaluation of cell viability of Hep55.1c cells after incubation with various concentrations of AuNCs and AP3-AuNCs in dark for 24 h, respectively. Error bars are expressed standard error with n=3.

Table S1. Characterization of the AP3-AuNC constructed in different solvents

Sample	Solvent	Size(nm)	Polydispersity index	Zeta potential
**AP3-AuNCs**	Ddw	137.0±51.1	0.194	-8.28
	80%EtOH	113±38.9	0.124	-9.46
	99%EtOH	168.9±72.6	0.205	-2.55
